# Oxidative-Stress-Mediated ER Stress Is Involved in Regulating Manoalide-Induced Antiproliferation in Oral Cancer Cells

**DOI:** 10.3390/ijms24043987

**Published:** 2023-02-16

**Authors:** Sheng-Yao Peng, Jen-Yang Tang, Ting-Hsun Lan, Jun-Ping Shiau, Kuan-Liang Chen, Jiiang-Huei Jeng, Ching-Yu Yen, Hsueh-Wei Chang

**Affiliations:** 1 Department of Biomedical Science and Environmental Biology, PhD Program in Life Sciences, College of Life Sciences, Kaohsiung Medical University, Kaohsiung 80708, Taiwan; 2 School of Post-Baccalaureate Medicine, Kaohsiung Medical University, Kaohsiung 80708, Taiwan; 3 Department of Radiation Oncology, Kaohsiung Medical University Hospital, Kaohsiung 80708, Taiwan; 4 Division of Prosthodontics, Department of Dentistry, Kaohsiung Medical University Hospital, Kaohsiung 80708, Taiwan; 5 School of Dentistry, College of Dental Medicine, Kaohsiung Medical University, Kaohsiung 80708, Taiwan; 6 Division of Breast Oncology and Surgery, Department of Surgery, Kaohsiung Medical University Hospital, Kaohsiung Medical University, Kaohsiung 80708, Taiwan; 7 Department of Oral and Maxillofacial Surgery, Chi-Mei Medical Center, Tainan 71004, Taiwan; 8 Department of Dentistry, National Taiwan University Hospital, Taipei 100225, Taiwan; 9 School of Dentistry, Taipei Medical University, Taipei 11031, Taiwan; 10 Center for Cancer Research, Kaohsiung Medical University, Kaohsiung 80708, Taiwan; 11 Cancer Center, Kaohsiung Medical University Hospital, Kaohsiung 80708, Taiwan

**Keywords:** marine sponges, ER stress, ER expansion, aggresome, apoptosis, autophagy, oral cancer

## Abstract

Manoalide provides preferential antiproliferation of oral cancer but is non-cytotoxic to normal cells by modulating reactive oxygen species (ROS) and apoptosis. Although ROS interplays with endoplasmic reticulum (ER) stress and apoptosis, the influence of ER stress on manoalide-triggered apoptosis has not been reported. The role of ER stress in manoalide-induced preferential antiproliferation and apoptosis was assessed in this study. Manoalide induces a higher ER expansion and aggresome accumulation of oral cancer than normal cells. Generally, manoalide differentially influences higher mRNA and protein expressions of ER-stress-associated genes (*PERK*, *IRE1α*, *ATF6*, and *BIP*) in oral cancer cells than in normal cells. Subsequently, the contribution of ER stress on manoalide-treated oral cancer cells was further examined. ER stress inducer, thapsigargin, enhances the manoalide-induced antiproliferation, caspase 3/7 activation, and autophagy of oral cancer cells rather than normal cells. Moreover, *N*-acetylcysteine, an ROS inhibitor, reverses the responses of ER stress, aggresome formation, and the antiproliferation of oral cancer cells. Consequently, the preferential ER stress of manoalide-treated oral cancer cells is crucial for its antiproliferative effect.

## 1. Introduction

Oral cancer is a severe type of cancer that is of increasing global concern [1]. The main factors correlated with mouth cancer include dangerous exposure to substance use, such as higher drinking, smoking, and betel nut chewing behaviors [2]. Additionally, viruses, fungus infections, nutritional deficiencies, radiation exposure, poor oral hygiene, and other chronic physical and chemical stimulations may be co-factors. In addition, gender, age, ethnicity, geographical culture, and lifestyle habits also affect the incidence of oral cancer [3]. There is also increasing research investment in the correlation between genes and environmental factors and the occurrence and deterioration of oral cancer [4]. Clinically, chemoradiotherapy and radiotherapy are used for oral cancer therapy, aside from surgery, but they occasionally generate adverse effects. Anti-cancer drugs with non-cytotoxicity that improve oral cancer therapy warrant continuous development.

Marine natural products are a diverse resource for the discovery of anti-cancer drugs [5,6,7,8]. Marine sponges are rich in many bioactive compounds [9,10,11], particularly for anti-cancer treatment. Manoalide is a sesterterpenoid, first isolated from a marine sponge *Luffariella variabilis* in 1980, and exhibits antibiotic [12], analgesic, and anti-inflammatory effects [13]. In 1993 and 2018, the repurposed application of manoalide for anti-cancer impacts was reported in murine lymphoma LI210 [14], cholangiocarcinoma, leukemia, and cervical cancer cells [15]. However, these studies [14,15] focused on isolating chemical compounds in the marine sponge, and they only provided IC_50_ values for anti-cancer evidence without investigating the detailed mechanisms.

The endoplasmic reticulum (ER) is a dynamic organelle that regulates diverse cellular functions, such as protein synthesis/folding, lipid synthesis/metabolism, and calcium storage/release, in response to cell stress and drug treatments [16,17]. ER stress is a condition in which many misfolding proteins are accumulated, which exceed the tolerance of protein refolding of ER. In turn, ER stress initiates the unfolded protein response (UPR). Heat-shock protein family A member 5 (HSPA5; GRP-78; BIP) is a master protein for ER stress regulation that connects to three primary UPR proteins, including protein kinase RNA-like ER kinase (EIF2AK3; PERK), inositol-requiring enzyme 1 alpha (ERN1; IRE1α), and activating transcription factor 6 (ATF6) [18]. Cellular stress causes ER dysfunction, such as protein immaturation and misfolding, which is a characteristic of the UPR [16]. Dramatic ER stress response generally triggers apoptosis [19,20] and autophagy [21,22] of cancer cells.

ER stress interplays with reactive oxygen species (ROS) in regulating redox signaling [23]. Disturbance of ROS homeostasis causes cellular and mitochondrial ROS accumulation, triggering ER stress [24,25]. Drugs with modulating functions for ROS and ER stress show potential effects in promoting cancer cell death [26,27]. For example, pendulone, a *Millettia dielsiana*-derived isoflavone, promotes apoptosis of lung cancer cells by upregulating ROS-dependent ER stress [28]. Several drugs showing modulation of ER stress have preferential antiproliferative effects on cancer cells without exhibiting cytotoxicity to normal cells [26]. Our previous reports demonstrate the detailed mechanism of manoalide in preferentially inhibiting the proliferation of oral cancer cells (which is not cytotoxic to normal cells), while upregulating cellular and mitochondrial ROS, DNA damage, and apoptosis [29,30].

Because ROS can induce ER stress as mentioned above, the ER stress function and the downstream responses of oral cancer cells treated with manoalide warrants a detailed assessment. This study aims to assess ER stress responses and their impact on apoptosis and autophagy of oral cancer cells under manoalide treatment. The modulating effects of oxidative stress induced by manoalide on ER stress of oral cancer cells are also investigated.

## 2. Results

### 2.1. ER Expansion Change by Manoalide: Oral Cancer vs. Normal Cells

**Figure 1:**
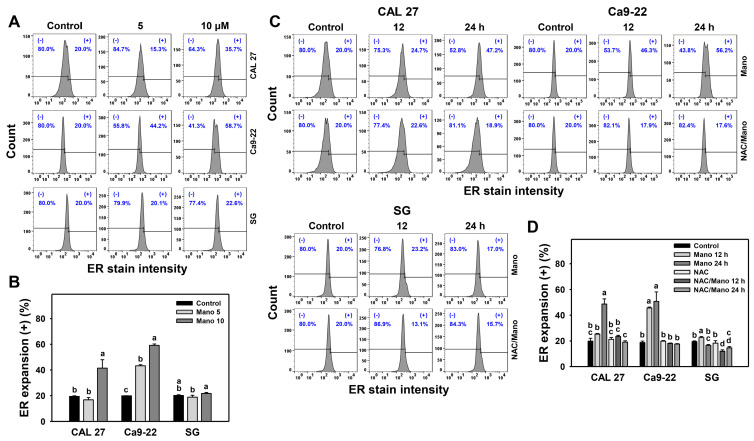
Manoalide causes ER expansion of oral cancer cells. (**A**,**B**) ER expansion analyses. Oral cancer (CAL 27 and Ca9-22) and normal oral (SG) cells received control, 5, and 10 μM manoalide treatments for 24 h. (+) was marked for ER expansion (+) (%). (**C**,**D**) NAC effects on time course changes of manoalide-induced ER expansion. Following NAC (10 mM for 1 h), cells received manoalide treatment (control and 10 μM) for 12 and 24 h, i.e., NAC/Mano. Data, means ± SDs (*n* = 3). Multiple comparisons were analyzed for the same cell lines. Treatments marked with different notes indicate a significant difference (*p* < 0.05 to 0.0001).

The changes in ER expansion patterns at different concentrations and exposure times of manoalide-treated oral cancer cells were assessed by flow cytometry (Figure 1A,C). Manoalide increased the intensity of ER (+) (%), namely ER expansion, in oral cancer cells (CAL 27 and Ca9-22) (Figure 1B,D). In contrast, ER expansion did not appear in normal cells (SG) because their ER staining intensities were similar to that of the control after manoalide treatment. Consequently, manoalide preferentially triggers ER expansion in oral cancer cells compared to normal cells.

Moreover, the influence of oxidative stress triggered by manoalide in controlling ER expansion was evaluated by the pretreatment of *N*-acetylcysteine (NAC) (Figure 1C). Manoalide-induced ER expansion of oral cancer cells was downregulated by NAC (Figure 1D). Hence, the role of ROS in manoalide-triggered ER expansion in oral cancer cells was confirmed.

### 2.2. Aggresome Change by Manoalide: Oral Cancer vs. Normal Cells

**Figure 2:**
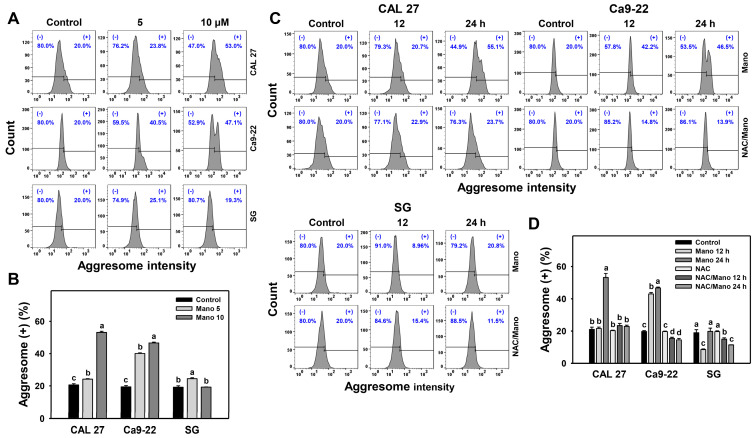
Manoalide causes the aggresome accumulation of oral cancer cells. (**A**,**B**) Aggresome analyses. Oral cancer and normal oral (SG) cells received control, 5, and 10 μM manoalide treatments for 24 h. (+) was marked for aggresome (+) (%). (**C**,**D**) NAC effects on time course changes of manoalide-induced aggresome. Following NAC (10 mM for 1 h), cells received manoalide treatment (control and 10 μM) for 12 and 24 h, i.e., NAC/Mano. Data, means ± SDs (*n* = 3). Multiple comparisons were analyzed for the same cell lines. Treatments marked with different notes indicate significant differences (*p* < 0.05 to 0.0001).

When ER stress is triggered, misfolded or denatured proteins are accumulated to form aggresomes [31]. The changes in aggresome generation patterns at different concentrations and exposure times of manoalide treatment in oral cancer cells were assessed by flow cytometry (Figure 2A,C). Manoalide increased the intensity of aggresome (+) (%) in oral cancer cells (Figure 2B,D). Consequently, manoalide preferentially triggers aggresome formation in oral cancer cells compared to normal cells.

Moreover, the presence of NAC estimated the oxidative stress involvement in modulating aggresome generation (Figure 2C). The manoalide-triggered aggresome accumulation of oral cancer cells was downregulated by NAC (Figure 2D). Hence, the role of ROS in manoalide-triggered aggresome formation in oral cancer cells was confirmed.

### 2.3. Modulation of ER Stress Signaling by Manoalide: Oral Cancer vs. Normal Cells

**Figure 3:**
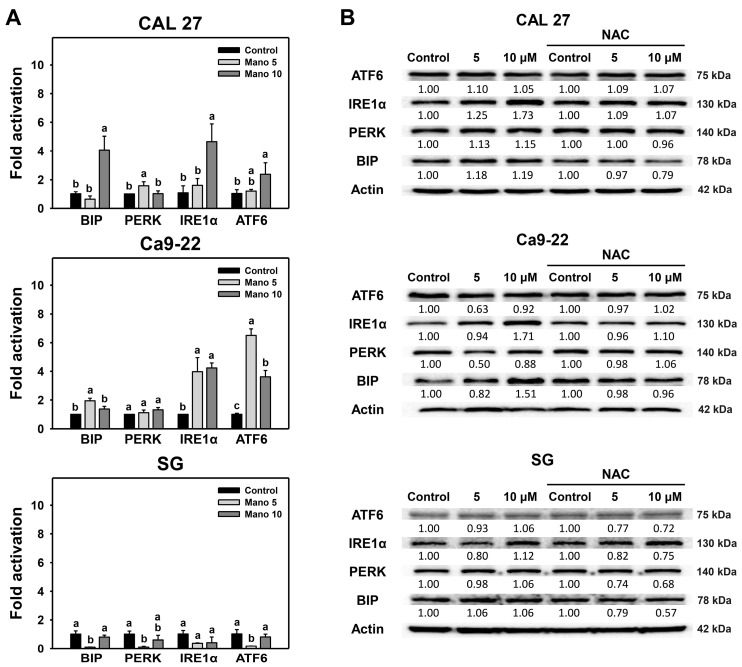
Manoalide causes ER stress gene expression of oral cancer cells. (**A**) Relative mRNA expressions (fold activation) of ER stress genes. Oral cancer and normal oral (SG) cells received control, 5, and 10 μM manoalide treatments for 24 h. Data, means ± SDs (*n* = 3). Multiple comparisons were analyzed for the same cell lines. For the same gene, treatments marked with different notes indicate a significant difference (*p* < 0.05 to 0.0001). (**B**) NAC effects on Western blotting for ER stress signaling. Following NAC (10 mM for 1 h), cells received manoalide treatment (control, 5, and 10 μM) for 24 h, i.e., NAC/Mano.

The mRNA expressions for ER stress signaling [32], including *BIP*, *PERK*, *IRE1α*, and *ATF6*, were assessed for manoalide treatment for 24 h in oral cancer cells and normal cells. The relative mRNA expression of these ER-stress-associated genes (*BIP*, *PERK*, *IRE1α*, and *ATF6*) in oral cancer cells was generally higher than in the control, while it showed no change in normal cells (Figure 3A). *BIP*, *IRE1α*, and *ATF6* genes were upregulated in oral cancer (CAL 27) cells at a high dose (10 μM). *IRE1α* and *ATF6* genes were overexpressed in oral cancer (Ca9-22) cells at 5 and 10 μM of manoalide. In contrast, the mRNA expressions of these ER-stress-associated genes of normal cells (SG) did not change.

Similarly, protein expressions for ER stress signaling were upregulated by manoalide in different oral cancer cells (Figure 3B). In CAL 27 cells, BIP, PERK, and IRE1α were moderately upregulated, and ATF6 was slightly regulated. In Ca9-22 cells, only BIP and IRE1α were moderately upregulated. In comparison, the protein expressions of these ER stress genes of normal cells (SG) were similar to the control and were lower than oral cancer cells. Notably, the moderately upregulated ER stress genes of manoalide treatment in oral cancer cells were downregulated by NAC. Consequently, manoalide preferentially triggers ER stress signaling in oral cancer cells compared to normal cells. Moreover, the role of ROS in manoalide-triggered ER stress signaling in oral cancer cells was confirmed.

### 2.4. Antiproliferation by Manoalide and ER Stress Inducer: Oral Cancer vs. Normal Cells

**Figure 4:**
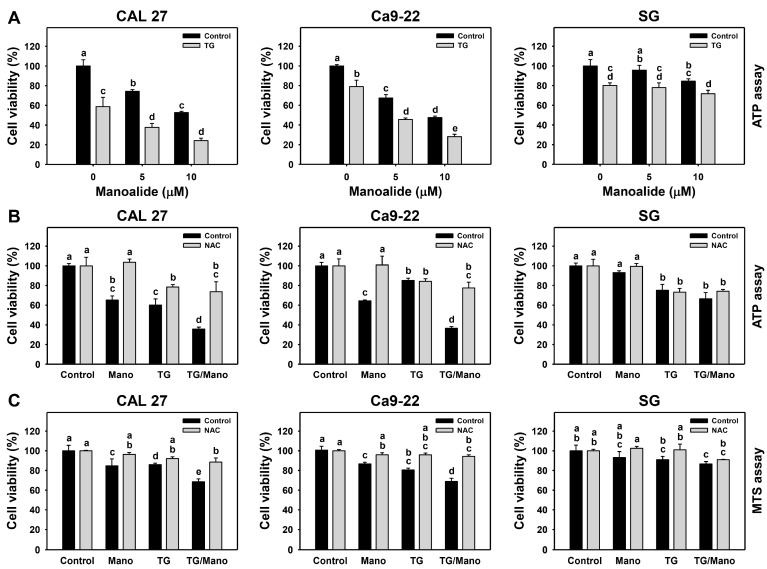
ER stress inducer (TG) enhances the antiproliferative effect of manoalide on oral cancer cells. (**A**) Cell viability of TG and/or manoalide was assessed by ATP assay. Cells were co-treated with TG (0.01 μM) and manoalide (0, 5, and 10 μM) for 24 h. (**B**,**C**) NAC effects on cell viability of TG and/or manoalide (Mano) were assessed by ATP and MTS assays. Following NAC (10 mM for 1 h), cells received TG and/or manoalide treatment (control, TG 0.01 μM, and/or Mano 10 μM) for 24 h. Data, means ± SDs (*n* = 3). Multiple comparisons were analyzed for the same cell lines. Treatments marked with different notes indicate a significant difference (*p* < 0.05 to 0.0001).

To investigate the proliferation impact of manoalide-triggered ER stress, cells were co-treated with the ER stress inducer thapsigargin (TG), followed by manoalide treatment. Their modulating abilities on the proliferation of oral cancer cells and normal cells were examined by ATP analyses because ATP content is a common cell viability indicator [33,34]. Under ATP-content-based cell viability, manoalide showed a preferential antiproliferative effect on oral cancer cells, while it showed minor changes in normal cells (Figure 4A). TG alone dramatically decreased the cell proliferation of oral cancer cells and slightly reduced it in normal cells. When the combined treatment of TG/manoalide was performed, the antiproliferative effect was enhanced in oral cancer cells. Furthermore, the combined treatment of manoalide/TG remains similarly viable to TG alone in normal cells (SG).

To explore the role of ROS, NAC was pretreated prior to the post-treatments of manoalide and/or TG in oral cancer cells and normal cells. Both ATP (Figure 4B) and MTS (Figure 4C) assays demonstrated the upregulated viabilities of manoalide and/or TG treatments in oral cancer cells upon NAC.

### 2.5. Modulation of Apoptosis by Manoalide and ER Stress Inducer: Oral Cancer vs. Normal Cells

**Figure 5:**
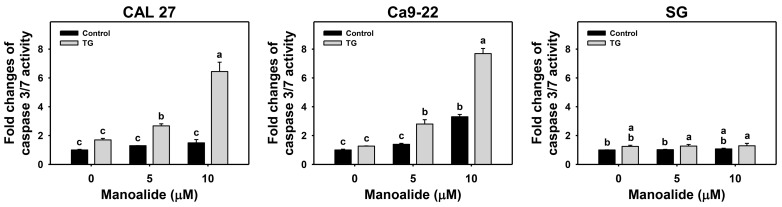
ER stress inducer (TG) enhances the caspase 3/7 activation of manoalide on oral cancer cells. Cells were co-treated with TG (0.01 μM) and manoalide (0, 5, and 10 μM) for 24 h. Data, means ± SDs (*n* = 3). Multiple comparisons were analyzed for the same cell lines. Treatments marked with different notes indicate a significant difference (*p* < 0.05 to 0.0001).

The impact of ER stress on manoalide-induced apoptosis was assessed by caspase 3/7 analyses. TG activated more caspase 3/7 activity in oral cancer cells than in the control. Moreover, TG/manoalide activated more caspase 3/7 activity than manoalide alone in oral cancer cells (CAL 27 and Ca9-22), particularly at a high dose (10 μM) (Figure 5). In contrast, the caspase 3/7 activity of manoalide treatment remained unchanged in normal cells (SG) with or without TG.

### 2.6. Modulation of Autophagy by Manoalide and ER Stress Inducer: Oral Cancer vs. Normal Cells

**Figure 6:**
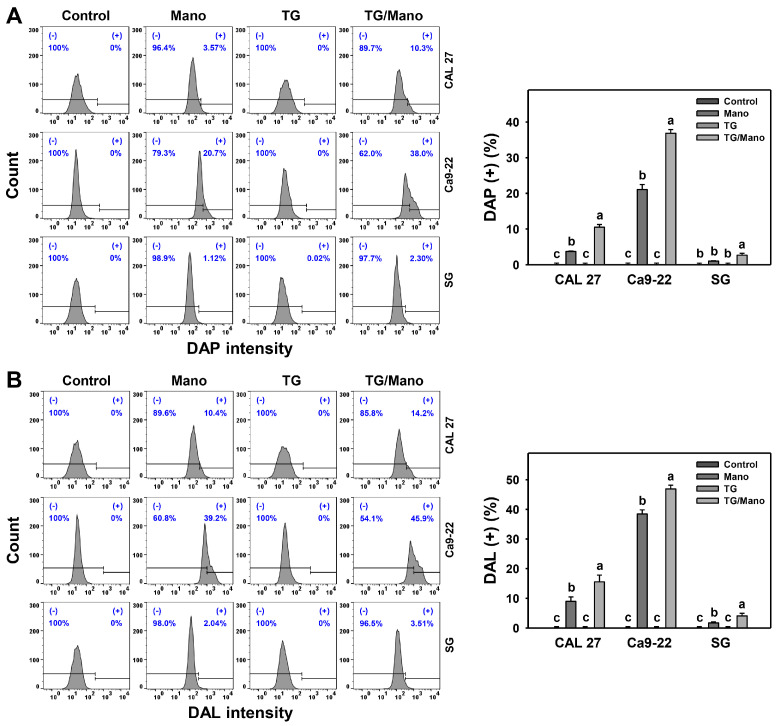
ER stress inducer (TG) enhances the autophagy of manoalide in oral cancer cells. (**A**,**B**) Autophagy change of TG and/or manoalide was assessed by DAPGreen and DALGreen assay. Cells were co-treated with TG (0.01 μM) or/and manoalide (10 μM) for 24 h. Data, means ± SDs (*n* = 3). Multiple comparisons were analyzed for the same cell lines. Treatments marked with different notes indicate a significant difference (*p* < 0.05 to 0.0001).

The impact of ER stress on manoalide-induced autophagy was assessed by DAPGreen (DAP) [35] and DALGreen (DAL) [36] analyses. Manoalide induced more DAP (+) and DAL (+) levels in oral cancer cells than in the control (Figure 6A,B). Moreover, TG/manoalide induced more DAP (+) and DAL (+) levels than manoalide alone in oral cancer cells (CAL 27 and Ca9-22) (Figure 6A,B). In contrast, the DAP (+) and DAL (+) levels of manoalide treatment remained unchanged in normal cells (SG) with or without TG.

## 3. Discussion

We previously found that manoalide provided preferential killing and the induction of oxidative stress, DNA damage, and apoptosis of oral cancer cells, while it showed minor changes to normal cells [29,30]. However, the interplay of ROS and apoptosis with ER stress [37] and the function of ER stress in the manoalide-caused antiproliferative effect of oral cancer were not investigated. The present study assessed the ER stress responses/signaling and apoptosis by modulating ROS or ER stress in the manoalide treatment of oral cancer cells and normal cells.

As detected by flow cytometry, manoalide at a high dose (10 μM) induced higher ER expansion and aggresome accumulation of oral cancer cells than normal cells (Figure 1 and Figure 2). These ER stress phenotypes were further examined by analyzing the mRNA and protein expression of ER stress signaling genes (Figure 3). CAL 27 and Ca9-22 cancer cells showed a higher mRNA expression of *BIP*, *IRE1α*, and *ATF6* genes than the untreated control, particularly at 10 μM manoalide, but these were slightly changed in normal cells. In comparison, manoalide did not induce *PERK* mRNA expression in oral cancer cells.

Similarly, the protein expressions of *BIP*, *PERK*, *IRE1α*, and *ATF6* genes of oral cancer cells and normal cells showed a similar tendency in their mRNA expressions. Notably, these ER-stress-associated proteins provided lower levels in normal cells than in oral cancer cells. This suggests that manoalide shows preferential induction of ER stress signaling in oral cancer, while it shows little changes in normal cells. However, the current study did not examine the impact of ER stress signaling on manoalide-triggered responses. It warrants a thoughtful assessment of knockdown *BIP* and/or *IRE1α* to see if the knockdown alters the outcomes of studies concerning viability, ER expansion, and apoptosis in the future.

Prolonged exposure to manoalide causes higher ER expansion and aggresome accumulation of oral cancer cells than normal cells, reversed by NAC. Similarly, some ER-stress-associated genes showing higher mRNA and protein expressions in oral cancer cells following manoalide treatment were reversed by NAC. This suggests that manoalide-triggered ER stress and its signaling are ROS-dependent. The role of ROS acting on apoptosis has been reported in our previous work [29,30], indicating that manoalide triggers apoptosis of oral cancer cells depending on ROS levels. Moreover, ER stress [21] and ROS [38] may trigger autophagy. This warrants a thorough investigation of the relationship between ROS, apoptosis, autophagy, and ER stress.

ER stress inhibits proliferation and triggers apoptosis of cancer cells [39,40,41,42]. TG, an ER stress inducer, suppresses the proliferation of breast cancer cells, and the combined treatment of TG with the engineered fusion protein (epidermal growth factor-proteolytic A subunit) enhances its antiproliferative effect [39]. TG also causes apoptosis of adrenocortical carcinoma [40] and prostate [41] cancer cells. Similarly, TG induces higher antiproliferation and apoptosis (caspase 3/7 activation) of manoalide-treated oral cancer cells than the control (Figure 4 and Figure 5). Like TG, manoalide triggers ER stress and apoptosis of oral cancer cells.

Furthermore, the impact of ER stress on manoalide-triggered antiproliferation, apoptosis, and autophagy was assessed in the present study. TG further enhances manoalide-induced antiproliferation, apoptosis (caspase 3/7 activation) (Figure 4 and Figure 5), and autophagy (Figure 6). In the example of hepatocytes, TG induces ER stress and antiproliferation, reversed by NAC [43], suggesting that TG triggers ROS induction to promote ER stress and antiproliferation. NAC can reverse the manoalide-induced antiproliferation [29] and ER stress responses (Figure 1, Figure 2 and Figure 3) of oral cancer cells. Similarly, NAC effects on the viability of TG/manoalide treatment of oral cancer cells were validated in the present study, demonstrating that TG/manoalide synergistically inhibited proliferation in an ROS-dependent manner (Figure 4B,C). Moreover, TG/manoalide synergistically triggered apoptosis (Figure 5), which may enhance antiproliferation. TG/manoalide also synergistically triggered autophagy (Figure 6); however, the role of autophagy in the antiproliferation of manoalide-treated oral cancer cells remains unclear. Consequently, manoalide exhibits the interplay of ER stress with ROS, apoptosis, and the preferential antiproliferation of oral cancer, but they are slightly changed in normal cells.

Besides apoptosis and autophagy, drug-induced ER stress may modulate and interact with several cellular functions, such as ferroptosis. For example, C2-ceramide triggers autophagy of liver cancer cells by upregulating oxidative stress and ER stress [44]. Notably, several anti-tumor drugs may exhibit the interplay between apoptosis and autophagy under ER stress [45,46,47]. Autophagy inhibitors promote apoptosis of prostate cancer cells triggered by overexpressing melanoma differentiation-associated gene 7 (mda-7) [48]. In contrast, downregulated apoptosis enhances autophagy after mda-7 overexpression. The relationship between ER-stress-modulated apoptosis and autophagy in manoalide-treated oral cancer cells may be explored by choosing their modulators. Moreover, ferroptosis triggers ER stress by interacting with apoptosis [49]. It warrants a thoughtful investigation for manoalide-induced other ER-stress-associated cell functions of oral cancer cells in the future.

## 4. Materials and Methods

### 4.1. Cell Culture and Chemicals

We chose the oral cancer cell lines (CAL 27 and Ca9-22) from the JCRB and ATCC for this study. As for normal oral cell line, we chose Smulow–Glickman (SG), a human normal gingival epithelial cell line that is well characterized [50,51] and commonly used to test drug safety for dental materials [52,53] and anti-oral-cancer drug development [54,55]. CAL 27/Ca9-22 and SG cells were cultured using mixtures of Dulbecco’s Modified Eagle Medium (DMEM) and F12 (Gibco, Grand Island, NY, USA) at ratios of 3:2 and 4:1 [29], respectively, with mixtures of 10% serum and common culture antibiotics.

Manoalide (CAYMAN CHEMICAL, Ann Arbor, MI, USA) and the ROS inhibitor NAC (Sigma-Aldrich, St. Louis, MO, USA) [56,57,58,59] were dissolved in dimethyl sulfoxide (DMSO) and 1X PBS. The DMSO concentration of all experiments (control and manoalide treatment) was the same for 0.1%. NAC at 10 mM was pretreated for 1 h before manoalide treatment. At 0.01 μM TG (Sigma-Aldrich), this ER stress inducer was co-treated by manoalide treatment.

### 4.2. ER Expansion

ER content was stable, but it increased (namely ER expansion) in response to ER stress. Using the Organelle-ID RGB^®^ III Assay Kit (Enzo Life Sciences, Farmingdale, NY, USA) [60], the degree of ER expansion was monitored by flow cytometry. Briefly, cells were mixed with ER staining dye under incubation for 30 min at 4 °C. After washing with the medium twice, cells were stood for 30 min at 37 °C and analyzed using the Guava easyCyte flow cytometer (Luminex, Austin, TX, USA), plotting with FlowJo software (Becton-Dickinson, Franklin Lakes, NJ, USA).

### 4.3. Aggresome

Upon ER stress, aggresomes commonly increase. Aggresomes were stained using Proteostat^®^ Aggresome Detection dye (Enzo Life Sciences) [61], and the degree of aggresome accumulation was determined by flow cytometry. Briefly, cells were prefixed with 4% paraformaldehyde for 30 min and permeabilized by 0.5% Triton X-100 for 30 min at 4 °C. Finally, cells were stained with aggresome staining dye (1:10,000) for 30 min, analyzed using a Guava easyCyte flow cytometer and plotted with FlowJo.

### 4.4. mRNA Expressions of ER-Stress-Associated Genes

RNA was extracted for cDNA conversion [62]. Quantitative RT-PCR (qRT-PCR) running with a touch-down PCR program was performed as described [62]. The mRNA expressions of four ER-stress-associated genes were analyzed [32], such as *BIP*, *PERK*, *IRE1α*, and *ATF6*. Their primer information is provided in Table 1. Glyceraldehyde 3-phosphate dehydrogenase (*GAPDH*) was the control gene [63,64]. The fold activation of mRNA expressions was evaluated by 2^-ΔΔCt^ calculations [65].

### 4.5. Western Blotting for ER-Stress-Associated Genes

The protein expressions of ER-stress-associated genes were detected by Western blotting by choosing their recognized antibodies, such as ATF6 (Abcam; 1:500), IRE1α, PERK, and BIP (Cell signaling, 1:1000), accompanied by the control antibody for β-actin (Sigma-Aldrich; 1:5000).

### 4.6. Cell Viability

Cell survival was readily detected by assessing intracellular ATP content (PerkinElmer Life Sciences, Boston, MA, USA) [66] and MTS assay (Promega Corporation, Madison, WI, USA) [67].

### 4.7. Caspase 3/7

Caspase 3/7 is the terminal caspase for apoptosis signaling. Caspase-Glo^®^ 3/7 commercial product (Promega, Madison, WI, USA) was used to detect the degree of caspase 3/7 activation [66]. Briefly, caspase-3/7 tetrapeptide DEVD substrates were equally mixed with a reaction buffer for 30 min incubation at 37 °C in darkness. Finally, the luminescence generated by activated caspase 3/7 was detected by luminometers (Berthold Technologies GmbH & Co., Bad Wildbad, Germany). The caspase 3/7 activity for each treatment was calibrated by its cell viability [68].

### 4.8. Autophagy

According to user instructions, autophagy-detecting dyes (Dojindo, Kumamoto, Japan), such as 12.5 nM DAPGreen [35] and 125 nM DALGreen [36], were chosen to probe autophagosomes/autolysosomes and autolysosomes, respectively. After PBS washing, drug-treated cells were detected and quantified by flow cytometry.

### 4.9. Statistics

One-way ANOVA with Tukey’s HSD post-hoc test was performed by JMP^®^12 (SAS Institute Inc., Cary, NC, USA) to determine the significances between multiple comparisons. Treatments of non-overlapping notes showed significant results.

## 5. Conclusions

Manoalide, characterized originally as an antibiotic, was here repurposed for anti-cancer treatments and the preferential antiproliferation and apoptosis of oral cancer cells was monitored, while it showed little impact on normal cells [29,30]. However, ER stress commonly interplayed with apoptosis, but the involvement of ER stress with oral cancer cells following manoalide treatment has rarely been investigated.

The present study validates that manoalide triggers more ER stress in oral cancer cells than in normal cells. ER expansion and the aggresome accumulation and gene expressions of ER-stress-associated proteins were upregulated by manoalide in oral cancer cells, but they were slightly changed in normal cells. Moreover, the potential connections between ER stress, antiproliferation, apoptosis, and autophagy were explored. Utilizing ER stress inducer TG, the manoalide-triggered antiproliferation and apoptosis were further enhanced. This finding indicates that manoalide-triggered ER stress is vital in controlling antiproliferation, apoptosis, and autophagy of oral cancer cells, but they showed low changes in normal cells. Consequently, manoalide showed preferential ER stress induction and contributes to the preferential antiproliferation of oral cancer cells.

## Figures and Tables

**Table 1:** Primers of ER-stress-related genes.

Genes	Forward Primers (5′→3′)	Reverse primers (5′→3′)	Accession Numbers
*BIP*	TGATCAAGATACAGGTGACCTGG	GTCTTTCACCTTCATAGACCTTGAT	NM_005347.5
*PERK*	TCATCCTCACAGGCAAAGGAAG	AGCCAATTCCCTATTGGGGA	NM_004836.7
*IRE1α*	AATTGTGTACCGGGGCATGT	TGCTCCACATACTCTTGCAGG	NM_001433.5
*ATF6*	ACAGAGTCTCTCAGGTTAAATCATG	GAGTTCCTGCTGATACTACTAGTGG	NM_007348.4
*GAPDH*	CCTCAACTACATGGTTTACATGTTCC	CAAATGAGCCCCAGCCTTCT	NM_002046.7

## Data Availability

Data are contained within the article.
